# Lateralization in Escape Behaviour at Different Hierarchical Levels in a Gecko: *Tarentola angustimentalis* from Eastern Canary Islands

**DOI:** 10.1371/journal.pone.0078329

**Published:** 2013-11-19

**Authors:** Enrique García-Muñoz, Catarina Rato, Fátima Jorge, Miguel A. Carretero

**Affiliations:** 1 CESAM, Centro de Estudios de Ambiente o do Mar, Universidade de Aveiro, Campus Universitário de Santiago, Aveiro, Portugal; 2 CIBIO, Centro de Investigação em Biodiversidade e Recursos Genéticos, Universidade do Porto, Campus Agrário de Vairão, Vairão, Portugal; 3 Departamento de Biología Animal, Biología Vegetal y Ecología, Universidad de Jaén, Campus de las Lagunillas, Jaén, Spain; Université Pierre et Marie Curie, France

## Abstract

At the individual level, to be behaviourally lateralized avoids costly duplication of neural circuitry and decreases possible contradictory order from the two brain hemispheres. However, being prey behaviour lateralized at higher hierarchical levels could generate different negative implications, especially if predators are able to make predictions after multiple encounters. These conflicting pressures, namely between the advantages for individuals and the disadvantages for populations could be concealed if higher-level lateralization would arise from the combination of lateralized behaviours of individuals which are mutually dependent. Here, we investigated the lateralization patterns in the escape behaviour of the gecko *Tarentola angustimentalis* undergoing a predatory attack simulation in a “T” maze experiment. Results showed that gecko populations displayed different degrees of lateralization, with an overall dominance of right-biased individuals. This trend is similar to that observed in the *Podarcis* wall lizards, which share predators with *Tarentola*. In addition, different morphological parameters plausible to affect refuge selection were explored in order to link directional asymmetries at morphological level with lateralization during refuge selection.

## Introduction

Brain lateralization has been explained in terms of avoidance of costly duplication of neural circuitry with the same function and decrease interference between different functions [1]. However, this could have crucial implications in terms of predator-prey interactions. In fact, different groups of species react faster to a predator approaching for the left side, which is controlled for the right hemisphere and known to control fear and escape responses [2], [3]. Following Brown et al. [4], the side of the behavioural bias could be fixed independently for every species because side is of secondary importance regarding the existence of functional specialization between the two brain hemispheres [5], [6]. This is due to the benefits associated with cerebral lateralization that are effective, regardless the directional bias at the individual level, but crucial at the population level [7]. Indeed, if individuals of a given species are lateralized, any predator will be facing an individually asymmetric prey. However, if preys are lateralized at a higher hierarchical level (population or species), predators could take advantage from earlier encounters with members of the same prey species and predict individual prey behaviour [7], [8].

Considering the close link between the habitat of an organism and its behaviour, performance, and morphology [9], it is expected that species are able to evolve/modify their morphology/behaviour in response to changes of the surrounding environment. The effect of evolution and environmental conditions on morphological directional asymmetries (MDA) and their influence on escape behaviour should be tested in order to explore the possible functional relationship between process and product [10]. Seligmann [11] reports an association of behavioural and morphological directional asymmetries at the level of individual lizards. On the other hand, behavioural experiments in fish [12] indicated that high predatory pressure could increase the proportion of lateralized individuals in a population, suggesting that predation pressure influences the development and evolution of lateralization in vertebrates.

The East Canary Gecko, *Tarentola angustimentalis*, is endemic to the Eastern Canary Islands (Spain) of Fuerteventura, Lanzarote and surrounding islets, being widely distributed from sea level to the highest points in both islands (807 and 670 m a.s.l., respectively) [13]. This thermophile gecko is found in a variety of habitats within its range including stony and rocky areas, lava fields, stone walls, sand dunes, saline plain with vegetation, scrublands, cultivated areas and human habitations. Among other predators, it is known to be part of the diet of the autocthonous birds *Tyto alba* and *Burhinus oedicnemus*, as well as of the introduced feral cats [14]. A case of predation by the syntopic lacertid lizard *Gallotia atlantica* has also been described [15] but this seems to be a rare event [16]. It is noteworthy that, contrary to what happens in other Canary Islands, the introduced feral cats are relatively scarce in Lanzarote and Fuerteventura [17], while no autochthonous terrestrial vertebrate predates geckos in these islands [14].

In the present study, we investigated the patterns of lateralization in five populations of *T. angustimentalis* inhabiting different habitats with the aim of analysing the occurrence of lateralized escape behaviour at different hierarchical levels, from individuals to the species level. Specifically, the objectives were: 1) to test the existence of a preference in the selection of left/right refuge in the escape behaviour for individuals, populations and the whole species; 2) in case of lack of lateralization at the population or the species levels, to determine whether this arises from individuals with no preference or from a mixture of right- and left-biased individuals; 3) to test whether left-right-biased individuals are equally common at the population and species level; 4) to test the effects of an inefficient predator pressure (resulting in tail regeneration) on refuge selection; 5) to test the occurrence of MDA at the population and species level and their correlations with lateralization in escape behaviour; and finally, 6) to examine the influence of minor MDA and test the developmental interpretation of the regression parameters.

## Materials and Methods

Collecting permits provided by the Cabildos Insulares (insular governments) of Fuerteventura (no. 2012018431) and Lanzarote (no. 2932) allowed collecting a maximum of ten individuals per population. This sample size has already been demonstrated to be sufficient to detect differences in lateralization trend among populations of *Podarcis* wall lizards [8]. Experiments were approved by the Committee of Animal Experimentation of the University of Porto (Portugal) under the Directive 2010/63/EU of the European Parliament. Thus, in October 2012, we collected by hand 50 adult geckos from five different populations of *T. angustimentalis*, corresponding to 10 adult individuals per population, two in Fuerteventura and three in Lanzarote. Regarding the two populations from Fuerteventura, Butihondo (28°07621′N, −14°30856′W, altitude 82 m a.s.l.) is an open habitat dominated by volcanic rocks with almost no vegetation and La Oliva (28°60749′N, −13°92563′W, 219 m a.s.l.) is an area with ancient agricultural walls and vegetation. As to the three localities in Lanzarote, La Caleta de Famara (29°06636′N, −13°58725′W, 219 m a.s.l.) is an open steppe with only herbaceous vegetation on sandy substrate and isolated volcanic stones; Nazaret-Teguise (29°04646′N, −13°56206′, 80 m a.s.l.) is an open area with hard substrate and scattered stones (mainly small) and very little vegetation and Yaiza (28.95179′N, −13.76788′W, 80 m a.s.l.) is a very open area of cultivated lands with large isolated stones and an some low agricultural walls.

**Figure 1:**
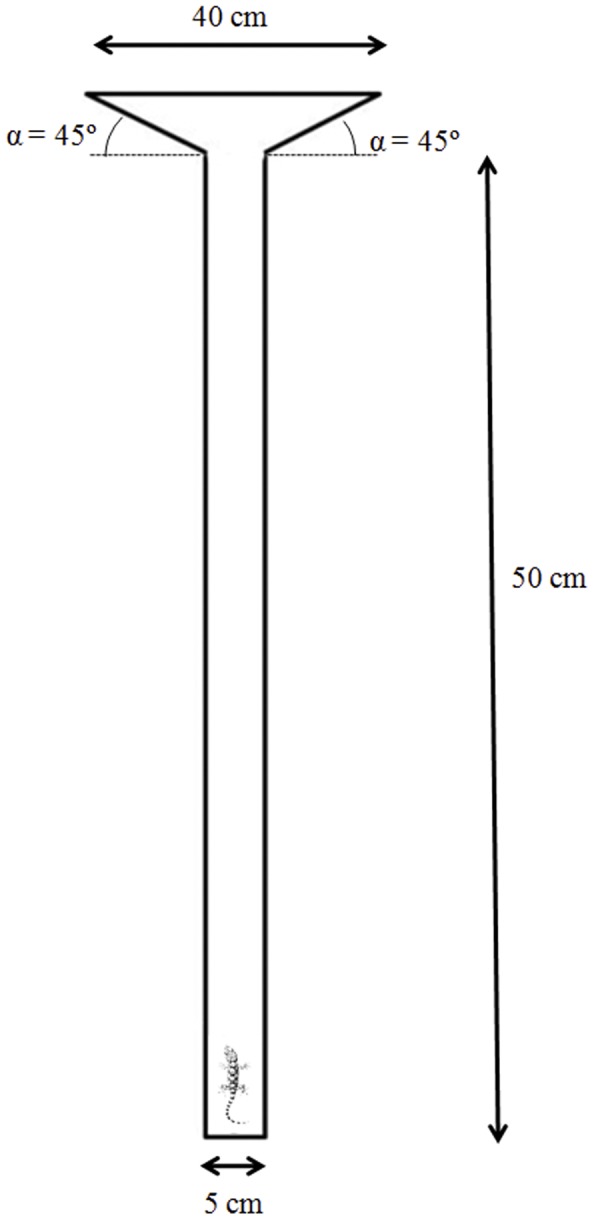
Schematic design of the experimental “T” maze used in the escape behaviour test (modified from García-Muñoz et al. 2012). Grey square represents the position of the video camera.

Geckos were carried to the laboratory, assigned with a code and kept in individual cloth bags. After the experiments, their snout-vent length (SVL) was measured to the nearest 0.01 mm with a digital calliper and tail state (original, O; regenerated, R) was registered. Once the whole procedure was concluded, all individuals were released at the original capture sites within 24 h. Food was not administrated to the animals during this short period. Room temperature (25–30°C) during the experiments was close to the activity temperatures described for other species of the genus *Tarentola* [18]. All experiments were carried out by night using an artificial light facing perpendicularly towards the maze. In each test, an individual gecko was placed in a 50×5×40 cm plexiglass experimental maze covered with a natural cork floor, providing excellent traction for running. No lid cover was added. Two refuges were attached to the extremes of the experimental maze, one on the right side and another on the left (Figure 1).

During each trial, the observer stayed in the back of the experimental maze, gently putting the gecko in the extreme opposite end of the refuges. The animal's escape was stimulated by beating a stick at the back of the experimental terrarium simulating a predator attack, using the right and left arm, alternately, to exclude influences in direction.

Each trial was filmed with a commercial video camera (Canon® Legria FS 200, lens 2.6–96.2 1∶2.0). Further analyses of the videos allowed assessing the side chosen by the geckos when escaping without ambiguity. Behaviours were categorised as left, right or neutral (when they went straight). In the rare cases when geckos stopped running before reaching either refuge of the experimental terrarium, the experimenter moved the stick again in order to stimulate the escape behaviour. Five consecutive tests per individual were conducted.

Furthermore, an indirect estimator of the predator effectiveness, the tail state, was recorded for each studied individual. Tail state was classified into two categories, original or regenerated, in order to test if previous (but far in time) unsuccessful predations may have an effect in refuge selection. Geckos with recent tail loss were excluded from the tests. In addition, different measurements of bilateral morphological characters (eye diameter, ED; forelimb length, FLL; arms vertical diameter, AVD; and arms horizontal diameter, AHD), that could be expected to have some influence in refuge side selection, were measured with a digital calliper (precision 0.01 mm). This was performed in order to detect for morphological directional asymmetries (MDA) and, if the case, to detect eventual links with refuge preference.

### Statistical analysis

Log-linear analysis was performed in order to detect interactions between categorical variables, namely, 1) Population (five states: Butihondo, La Oliva, La Caleta de Famara, Nazaret-Teguise and Yaiza); 2) Tail State (two states: O, original; R, regenerated); 3) Trial (five consecutive trials per individual); and 4) Refuge Side (three states: left, neutral or right).

Furthermore, for compiling the responses to repeated tests of the same individual we used the laterality index (LI), modified from [8] calculated as: frequency of right runs−frequency of left runs/(total frequency of right runs+left runs+neutral runs). Values of LI lower or greater than 0 indicate a left (−1) or right preference (+1), respectively, whereas a 0 value indicated no preference. In addition, a binomial test (Fisher exact P, two-tailed) was employed to test the left or right refuge preference compared with theoretical no preference, at individual, population and species levels. Finally, to test if left and right-type individuals are equally common at the population and the *T. angustimentalis* levels, a binomial test (Fisher exact P, two-tailed) was carried out. In parallel, LI was also used as a continuous dependent variable to feed a general linear model with population and tail state as factors.

Different t-tests for dependent samples were used in order to compare differences in right and left (left, x-axis; right, y-axis) measurements (log-transformed) in the four morphological characters studied, to test the null hypotheses, H_0_: right = left. In addition, directional asymmetries (DA) at the species level were quantified with the regression parameters *a* (constant) and *b* (slope) of the regression between left and right side (see 10, 19). These authors interpreted these parameters as follow: the regression constant *a*, significantly different from zero, indicates which side begins first to develop. On the other hand, the slope *b* indicates which side develops earlier, when significantly different from 1. This way, MDA describes the observed phenotype, while directional trajectories describe the components, *a* and *b* of a process from which results the phenotype. To prevent pseudoreplication, regression parameters were calculated over the population means of left and right parts.

The software STATISTICA 10 [20] (Statsoft Inc. 2011) and *p*<0.05 significance level were used in all statistical analysis.

## Results

Log-linear analyses showed significant differences in frequency of original versus regenerated tails, refuge side preferences, Population*Tail, and refuge side preferences, Population*Side, but no for other interactions (Table 1).

**Table 1:** Results of log-linear analysis to detect interactions between 1) Population (5: Butihondo, La Oliva, Caleta de Famara, Nazaret-Teguise and Yaiza), 2) Tail state (Tail, 2: original or regenerated), 3) Trial (5 trials per individual), and 4) Refuge Side (3: Left, Neutral, Right).

		Partial association		Marginal Association	
	df	Chi-square	*p*	Chi-square	*P*
**Population**	4	<0.0001	1.000	<0.0001	1.000
**Tail**	1	11.141	**0.001**	11.141	**0.001**
**Race**	4	<0.0001	1.000	<0.0001	1.000
**Side**	2	35.000	**0.000**	35.000	**0.000**
Population*Tail	4	18.843	**0.001**	17.630	**0.001**
Population*Trial	16	0.238	1.000	<0.0001	1.000
Population*Side	8	20.310	**0.009**	18.872	**0.016**
Tail*Trial	4	0.024	1.000	<0.0001	1.000
Tail*Side	2	1.836	0.399	0.613	0.736
Trial*Side	8	7.414	0.493	7.166	0.519
Population*Tail*Trial	16	0.863	1.000	<0.0001	1.000
Population*Tail*Side	8	12.498	0.130	12.432	0.133
Population*Trial*Side	32	16.248	0.991	18.116	0.977
Tail*Trial*Side	8	7.235	0.512	8.819	0.358

Model: Population*Side; Chi-square_135_ = 89.097, *p* = 0.999. (*p* values lower than 0.05 are marked in bold).

**Figure 2:**
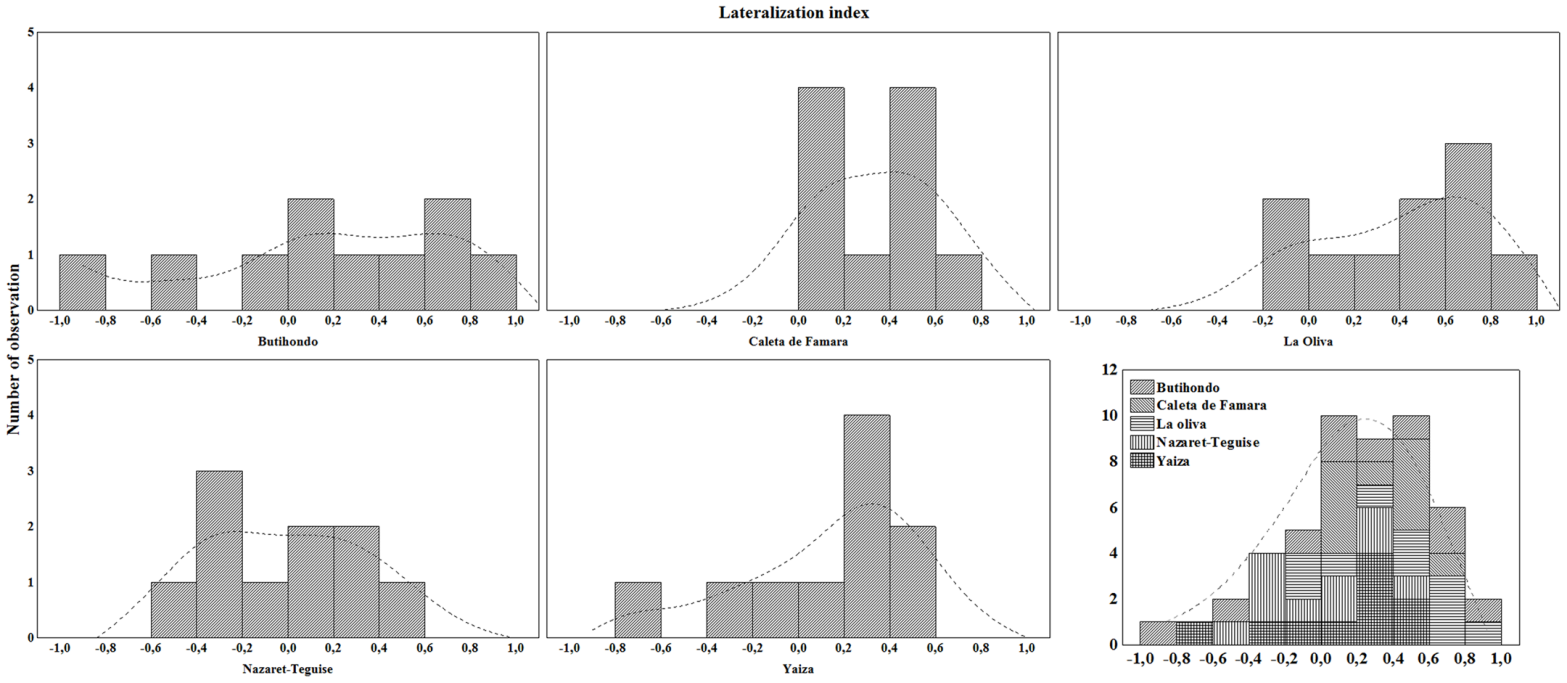
Frequency plots showing the number of observations of each value plotted on the X axis. The X axis is the Lateralization Index (LI) for each individual, where LI = (right−left)/(right+neutral+left). Fisher exact test *p* two-tailed was used in order to test the null hypothesis: no refuge preference (see also Table 2).

At the *T. angustimentalis* level (i.e. species level), the overall sample of 50 individuals used in this experiment showed a LI = 0.3, indicating neutral, marginally right refuge skew (Table 2, Figure 2). At the population level, only La Caleta de Famara and La Oliva populations showed right refuge preference, while the remaining three (Butihondo, Nazaret-Teguise and Yaiza) showed no refuge preference.

**Table 2:** Number of lateralized (L: Left; R: Right) and non lateralized (N: Neutral) individuals found in each population.

	L	N	R	LI	Left %	Right %	*p*
**Butiondo**	1	6	3	0.2	28%	72%	**<0.01**
**Caleta de Famara**	0	5	5	0.5	13%	87%	**<0.001**
**La Oliva**	0	4	6	0.6	8%	92%	**<0.001**
**Nazaret-Teguise**	0	9	1	0.1	41%	59%	>0.05
**Yaiza**	1	7	2	0.1	10%	90%	**<0.001**
**Species Level**	2	31	17	0.3	22%	78%	**<0.001**

Lateralization Index (LI) = (right runs−left runs)/(right runs+neutral runs+left runs). Left% and Right% represent the percentage of times that all the lateralized individuals (left and right) chose the left or right refuge. Fisher exact test P two-tailed was used only with lateralized individuals, in order to test if left- and right- lateralized individual were equally common at population or species level (see also Figure 2).

At the individual level, 19 of the tested individuals (38%) showed refuge preference, while 31 (62%) showed no refuge preference. Right lateralization was dominant amongst the lateralized individuals (two individuals were left lateralized while 17 individuals were right lateralized; Fisher exact test two tailed, *P* = 0.013).

ANOVA showed differences in SVL (log-transformed) between populations (F(4, 45) = 13.93; P<0.001). Although, SVL had no significant effect on LI at the species level (r2 = 0.0067; p = 0.572), the populations from Butihondo (r2 = 0.512; r = 0.716; p = 0.01) and La Oliva (r2 = 0.386; r = −0.621; p = 0.05) showed a significant interaction but with inverse relationship: while in Butihondo population the LI increased proportionally to the SVL, in La Oliva the LI decreased when SVL increased. In the other three populations no significant effects were detected.

**Figure 3:**
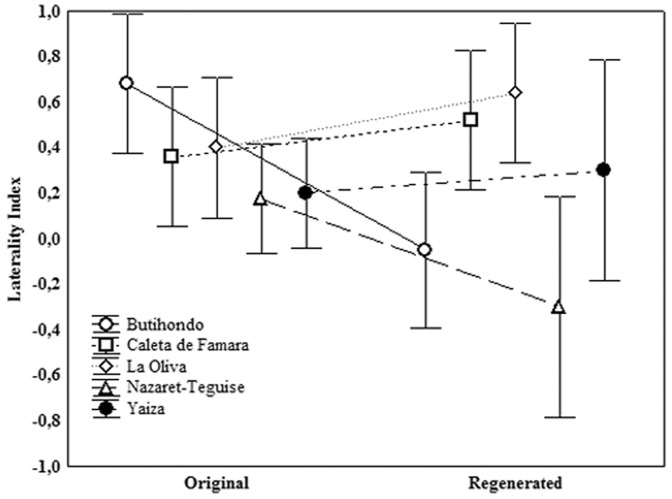
Laterality index showed in the five population studied, classified in function of the tail state (original vs regenerated).

The General Linear Model, performed using LI as depended variable, showed that *tail state* has no influence in refuge selection at the species level. However the interaction population*tail state was significant (Table 3, Figure 3). Namely, individuals from Butihondo with regenerated tails showed a left refuge preference while individuals with original tails showed a right preference (post-hoc Duncan's test, *p* = 0.01). A similar trend appearing in the population of Nazaret-Teguise lacked statistical support (post-hoc Duncan's test, *p* = 0.06).

**Table 3:** Results of General Linear Model (GLM) that analyzes the effects of Tail state (Tail; original vs regenerated) and Population (5, Butihondo, La Oliva, Caleta Famara, Nazaret-Teguise, Yaiza) on laterality index, LI = (right run−Left run)/(right+left run).

	df	F	p
**Intercept**	1	28.911	0.000
**Population**	4	3.318	0.019
**Tail**	1	2.130	0.152
Population*Tail	4	4.227	0.006
**Error**	40		

The t-test for dependent samples showed that only FLL differed between right and left side in four out of five populations studied (excepting Butihondo), with the left forelimb being longer than the right one (Table 4). The remaining morphometric variables displayed no significant MDA. At the species level the left bias persisted (Table 4). The regression parameters between left FLL (x-axis) and right FLL (y-axis) (log-transformed) obtained from the means of FLL left and right at population level were *a* = 0.2021 for the constant and *b* = 0.6971 for the slope. The latter significantly differed from 0 and 1 respectively, indicating that the right side begins first to develop (constant *a*), while *b* (slope) indicates that the left side develops faster (r = 0.8773; p = 0.05). At population level the (significant) regression parameters were: Butihondo, *a* = 0.345, *b* = 0.739, p = 0.05; Caleta de Famara, *a* = 0.032, *b* = 0.967, p<0.001; La Oliva, *a* = −0.107, *b* = 1.059, p<0.001; Nazaret-Teguise, *a* = −0.087, *b* = 1.044, p<0.001; Yaiza, *a* = 0.285, *b* = 0.786, p<0.001.

**Table 4:** T-test for dependent samples in all the morphological character studied (arm vertical length, AVL; arms horizontal length, AHL; forelimb length, FLL; eye diameter, ED).

	AVL	AHL	FLL	ED
**Butihondo**	(2.46±0.45; 2.30±0.35) R = L	(2.94±0.37;2.85±0.53) R = L	(21.27±1.77;21.26±1.91) R = L	(3.45±0.36;4.10±0.32) R = L
	ns	ns	ns	ns
**Caleta de Famara**	(3.60±0.91;3.69±0.40) R = L	(4.33±0.65;4.24±0.65) R = L	(27.47±2.56;28.47±2.62) R<L	(4.66±0.29;4.49±0.21) R = L
	ns	ns	(t = 3,189; p<0.01)	ns
**La Oliva**	(3.07±0.52;3.09 ±0.61) R = L	(3.72±0.42;3.60±0.71) R = L	26.18±2.63;27.52±2.36) R<L	(4.61±0.22;4.73±0.16) R = L
	ns	ns	(t = 3,502; p<0.001)	ns
**Nazaret-Teguise**	(3.42±0.40;3.79±0.42) R<L	(4.08±0.34;4.24±0.39) R = L	(26.27±2.16;27.22±2.13) R<L	(4.67±0.36±4.62±0.43) R = L
	(t = 2,632; p<0,05)	ns	(t = 7,655; p<0.001)	ns
**Yaiza**	(2.96±0.49;3.24±0.50) R = L	(3.80±0.69;3.88±0.47) R = L	(24.47±3.54;25.25±3.21) R<L	(4.45±0.33;4.66±0.41) R<L
	ns	ns	(t = 2,472; p<0.05)	(t = 2,732;p<0.05)
**Species level**	(3.11±0.62;3.24±0.69) R = L	(3.79±0.68;3.78±0.74) R = L	(25.21±3.12;26.14±3.50) R<L	(4.48±0.41;4.53±0.36) R = L
	ns	ns	(t = 5,679; p<0.001)	ns

The null hypothesis (R = L; p>0.05) was rejected at p-values lower than 0.5. In addition the mean value (right length±sd; left length ±sd) and the relation between right and left side are shown.

No significant correlations between FLL and LI were found.

## Discussion

Our results show that escape behaviour differed in the refuge side preferences between populations of the same gecko species. Populations from La Caleta de Famara (Lanzarote) and La Oliva (Fuerteventura) showed a right-side refuge preference, arising from the dominance of neutral and right-biased individuals and the absence of left-biased individuals. By contrast, populations from Butihondo (Fuerteventura), Nazaret-Teguise and Yaiza (Lanzarote) showed no refuge preference. In these three populations, the lack of lateralization arose either from a majority of individuals with no preference or right-biased (e.g. Nazaret-Teguise) or from a combination of right- neutral- and left- biased individuals (e.g. Butihondo and Yaiza). According to Brown et al. [4], this heterogeneity would be derived from different predator-prey interactions between populations. These authors found that a high predatory pressure could be important in increasing the number of lateralized individuals in a population. Nevertheless, log-linear analyses showed that the interactions Population*Tail*Side and Tail*Side were not significant. Although the GLM detected statistical differences between populations and tail states and the post-hoc tests pointed these differences in refuge preference, for the population from Butihondo, these results need to be interpreted with caution since in this population the lack of lateralization arose from a mix of individuals in each category of the LI.

In squamate reptiles, tail autotomy is an antipredator mechanism, which was initially used as a proxy for the frequency of predation [21], [22] but more recently reinterpreted as inefficient predation [23], [24], [25]. Apparently, events of inefficient predation which had taken place far back in the past had no systematic effects on the lateralized escape behaviour of these geckos. Effects of efficient predation, not reflected by regenerated tails, cannot be discarded since geckos could be negatively selected (by predation of the whole animal) on the basis of their antipredatory behaviour compared to conspecifics of the same population. However, the relatively low predation pressure undergone by lizards in insular ecosystems [26] might make this phenomenon less important than for their continental relatives.

At lower levels, the lateralized individuals displayed a strong preference for the right refuge. Recent studies conducted with the wall lizards, *Podarcis muralis* [27], [28] and *Podarcis hispanica* sensu lato [8], showed that these lizards predominantly monitored a predator with the left eye while escaping preferably to the right refuge. Since both *Podarcis* wall lizards and *Tarentola* geckos are separated by more than 150 Mya of independent evolution [i.e 29], this bias might well reflect a general trend in the development of vertebrates, but this should be corroborated by wider comparative studies. Being lateralized at an individual level could provide an advantage for individual geckos to be more effective in escaping from a predator's attack by enabling separate and parallel processing to take place in the two hemispheres (left eye—right hemisphere controlling the predator; right eye—left hemisphere searching for a refuge). However, in a population or species context [8], if most individuals [8], [27], [28] are biased in the same direction (in this study, right), their behaviour will become more predictable to predators [30], resulting in a disadvantage for those individuals following the most frequent behaviour. On the other hand, higher-level lateralization may eventually arise when the fitness of an individual asymmetrical organism depends on what other individual asymmetrical organisms do.

In *Tarentola angustimentalis* most populations showed no signs of lateralization. This could suggest the existence of higher levels of (efficient) predation in these sites compared to La Oliva and La Caleta de Famara where individuals seem to be right-biased. However, this should be validated by an independent estimation of predation pressures on each population [30], for which additional fieldwork would be necessary. In addition, although SVL has no significant effects on LI at the species level, it is expected that bigger individuals have a longer history of predator encounters, recommending an investigation of behavioural lateralization across age classes.

Finally, an association between behavioural lateralization and morphological asymmetry has already been reported for an insular gecko population from the genus *Hoplodactylus* [11]. In this study the first hindlimb lifting from the substrate by most geckos was the one possessing the lowest number of subdigital lamellae. Although we did not record such trait, the prediction would be that a positive correlation of number of lamellae and escape side occurs, and this could be a link between functional causes and the association of morphological and behavioural asymmetries. Alternatively, our own results showed that left forelimb length was longer than right in four of the five populations studied and for the whole sample. In addition, at the species level the same significant trend was found, thus *T. angustimentalis* showed longer left FL than the right one. These results are in accordance with the “pivot hypothesis” presented in [11], according to which differences in size between both forelimbs should be linked with the facilitation in turning. The shorter forelimb would be the internal point where the turn starts, while the larger forelimb should be the external point during the turn. Nevertheless, the comparisons between *Hoplodactylus* and *Tarentola* may be problematic, not only because they differ in reproductive modes (viviparous vs. oviparous, respectively) but mainly because they belong to highly divergent evolutionary lineages within the Gekkota (Diplodactylidae and Phyllodactylidae) separated by more than 100 Mya of independent evolution [31], [32], including the independent acquisition of toe pads [33].

Here, no significant correlations were found between morphological asymmetries and behavioural lateralization (estimated by LI). Nevertheless, according to previous studies [10], [19], the directional trajectory (DT) showed by the analysis of the regression parameters, *a* (constant) and *b* (slope), indicates that the right side begins first to develop, while the left side develops fastest. In the light of the present results, new experimental studies should be conducted to fully understand the links of escape behaviour with MDA (plasticity) and DT (evolutionary history) elucidating the subjacent proximal (developmental mechanisms) and ultimate (functional) causes.
